# Treatment-Mediated Alterations in HIV Fitness Preserve CD4^+^ T Cell Counts but Have Minimal Effects on Viral Load

**DOI:** 10.1371/journal.pcbi.1001012

**Published:** 2010-11-24

**Authors:** Naveen K. Vaidya, Libin Rong, Vincent C. Marconi, Daniel R. Kuritzkes, Steven G. Deeks, Alan S. Perelson

**Affiliations:** 1 Theoretical Biology and Biophysics Group, Los Alamos National Laboratory, Los Alamos, New Mexico, United States of America; 2 Department of Mathematics and Statistics, Oakland University, Rochester, Michigan, United States of America; 3 Emory University School of Medicine, Division of Infectious Diseases, Atlanta, Georgia, United States of America; 4 Section of Retroviral Therapeutics, Brigham and Women's Hospital and Division of AIDS, Harvard Medical School, Boston, Massachusetts, United States of America; 5 Department of Medicine, University of California, San Francisco, San Francisco, California, United States of America; 6 San Francisco General Hospital, San Francisco, California, United States of America; Imperial College London, United Kingdom

## Abstract

For most HIV-infected patients, antiretroviral therapy controls viral replication. However, in some patients drug resistance can cause therapy to fail. Nonetheless, continued therapy with a failing regimen can preserve or even lead to increases in CD4^+^ T cell counts. To understand the biological basis of these observations, we used mathematical models to explain observations made in patients with drug-resistant HIV treated with enfuvirtide (ENF/T-20), an HIV-1 fusion inhibitor. Due to resistance emergence, ENF was removed from the drug regimen, drug-sensitive virus regrown, and ENF was re-administered. We used our model to study the dynamics of plasma-viral RNA and CD4^+^ T cell levels, and the competition between drug-sensitive and resistant viruses during therapy interruption and re-administration. Focusing on resistant viruses carrying the V38A mutation in gp41, we found ENF-resistant virus to be 17±3% less fit than ENF-sensitive virus in the absence of the drug, and that the loss of resistant virus during therapy interruption was primarily due to this fitness cost. Using viral dynamic parameters estimated from these patients, we show that although re-administration of ENF cannot suppress viral load, it can, in the presence of resistant virus, increase CD4^+^ T cell counts, which should yield clinical benefits. This study provides a framework to investigate HIV and T cell dynamics in patients who develop drug resistance to other antiretroviral agents and may help to develop more effective strategies for treatment.

## Author Summary

The impact of antiretroviral drug-resistance on viral load, CD4^+^ T cells, and clinical outcomes is complex. We used mathematical models to evaluate the benefits of HIV drug therapy in the presence of drug-resistant virus. As an example, we considered resistance to enfuvirtide, the first FDA-approved fusion inhibitor. If viral load increases on drug therapy due to drug resistance, therapy with this drug may be stopped. We found that the drug resistant virus is less fit than the drug-sensitive virus in the absence of drug, and this fitness disadvantage causes the loss of drug-resistant virus during drug interruption. After the drug-sensitive virus replaces resistant virus, enfuvirtide therapy was re-administered. Analyzing the resulting viral kinetics, we demonstrate that despite the inability of the re-administered drug to suppress viral load because of the continued presence of drug resistant virus, therapy still provides benefit to the patient by preserving or increasing peripheral blood CD4^+^ T cell levels.

## Introduction

Antiretroviral therapy has been used to successfully treat HIV-1 infection. However, a subset of patients develops drug resistance followed by an observable increase in plasma HIV viral load. This “virological failure” usually triggers a change in the drug regimen. Here we examine a situation in which patients had developed resistance to most common drugs and a novel agent, enfuvirtide, was added to their failing drug regimen. When resistance to enfuvirtide developed the use of this agent was discontinued in the hope that drug-sensitive virus would outcompete the resistant virus and enfuvirtide could be given again. Despite the fact that resistance developed when enfuvirtide was re-administered and viral loads were unable to be suppressed, CD4^+^ T cell counts were preserved or increased. Observing increasing CD4^+^ T cell counts without viral suppression is intriguing and suggests that issues of viral fitness may play a role. Fitness costs have been associated with drug resistance not only to enfuvirtide but also to other drug classes [1]–[6]. Further, despite virologic failure due to the emergence of drug resistance, continued treatment that imposes selective pressure on drug sensitive virus and causes outgrowth of resistant HIV is often associated with benefits such as higher sustained CD4^+^ T cell counts and reduction in the risk of morbidity and mortality [2]–[5]. To uncover the nature of the CD4^+^ T cell increase and to determine a general principle that may be useful in developing treatment strategies in the face of drug resistance, we performed a detailed viral kinetic analysis of a set of patients treated with enfuvirtide in which longitudinal measurements of drug sensitive and drug resistant viral levels, as well as CD4 counts, were available.

Enfuvirtide (ENF), formerly called T-20, is a 36 amino acid synthetic peptide that binds to the HR-1 region of the HIV-1 gp41 molecule, thereby preventing fusion of the viral membrane with the target cell membrane [7]. It is the first FDA-approved HIV-1 fusion inhibitor [8]. As ENF is expensive and must be administered parenterally, it is often reserved for heavily pretreated patients with limited therapeutic options [9]–[13]. ENF acts extracellularly prior to viral entry. This feature provides a number of benefits, such as less susceptibility to cellular efflux transporters that lower the effective intracellular concentrations of other classes of antiretroviral drugs and little or no drug-drug interactions with drugs metabolized by the CYP 450 or N-acetyltransferase route [14].

As with other antiviral drugs, in patients treated with ENF, the high replication rate of HIV and the low fidelity of HIV reverse transcriptase can lead to the development of drug resistance [14]. Resistance to ENF occurs due to amino acid substitutions within the HR-1 region of gp41 at amino acids 36–45 of HIV-1 gp41 with G36D, G36S, G36V, G36E, V38A, V38M, V38E, Q40H, N42T, and N43D being the most common ENF resistant mutations [12], [13], [15]. These mutations result in significantly reduced binding of ENF to HR-1 [16]. Since ENF is expensive and poorly tolerated, many individuals interrupt this drug once virologic failure is confirmed. In a single arm prospective study of individuals exhibiting virologic failure on ENF, selective interruption of ENF was not associated with any appreciable increase in HIV RNA levels, suggesting that the drug had only limited residual activity and hence its use during failure may not be warranted [11], [17]. Observational data from other groups, however, have suggested that there may be a CD4^+^ T cell benefit associated with certain ENF-associated mutations [18]. These data suggest that despite virological failure the drug may have continued benefit due to alterations in the virus's pathogenic effects.

Interruption of ENF in individuals with ENF-resistance is associated with a rapid decay in the resistant variant [11], [13], [17]. The reason resistant virus decays in the absence of drug is not fully understood. Although the rebound of archived more “fit” wild-type virus is often cited as the major mechanism whereby HIV resistance decays in the absence of therapy [13], [17], ongoing evolution within the envelop gene and the eventual selection of the wild-type virus may also account for the loss of ENF resistance when this drug is interrupted [9].

Despite marked differences in fitness of drug-sensitive and drug-resistant viruses and evidence of ongoing viral evolution, plasma HIV-1 RNA levels remain almost constant during ENF interruption [13]. This apparent paradox suggests that viral fitness may not be a major determinant of the steady-state level of viremia. To more fully understand the role of viral fitness as well as other parameters determining the dynamics of HIV-1 during ENF interruption, we use mathematical models to study the competition between ENF sensitive and ENF resistant viruses after the interruption of ENF and during subsequent re-administration. We consider only the V38A mutant because this single substitution in HIV-1 gp41 is the most frequently observed in drug resistant virus [19] and data on the population size of mutants with V38A are available [13]. We estimate the rate of forward and backward mutations, the replication capacity of both drug-sensitive and drug-resistant viruses, and the efficacy of ENF against viral fusion when it is re-administered after interruption. We also examine the effect of target cell level on the dynamics and steady states of drug sensitive and resistant viruses during ENF interruption and subsequent re-administration. Lastly, we discuss virus population turnover and plasma viral RNA levels during the presence and absence of the drug.

## Methods

### Patient Data

We obtained wild-type and V38A mutant viral load and CD4^+^ T cell data from Department of Medicine, University of California-San Francisco, CA, USA, San Francisco General Hospital, San Francisco, CA, USA and Section of Retroviral Therapeutics, Brigham and Women's Hospital and Division of AIDS, Harvard Medical School, Boston, MA, USA. Viral load and CD4^+^ data were obtained for three HIV-1 infected subjects (P1, P2, and P3) during ENF interruption who continued to receive the other drugs in their antiretroviral regimen. Before ENF interruption, subjects P1, P2 and P3 were treated with ENF for 27, 33 and 39 weeks, respectively, and each of them had the V38A mutation as the predominant virus population (more than 85% frequency). Viral load and CD4^+^ data were also obtained during subsequent 4-week re-administration of ENF after interruption for 76, 68 and 38 weeks, respectively. For subject P3, the data were also collected during a second interruption of ENF. Therefore, there were two data sets during ENF interruption for subject P3.

### Mathematical Model

**Figure 1:**
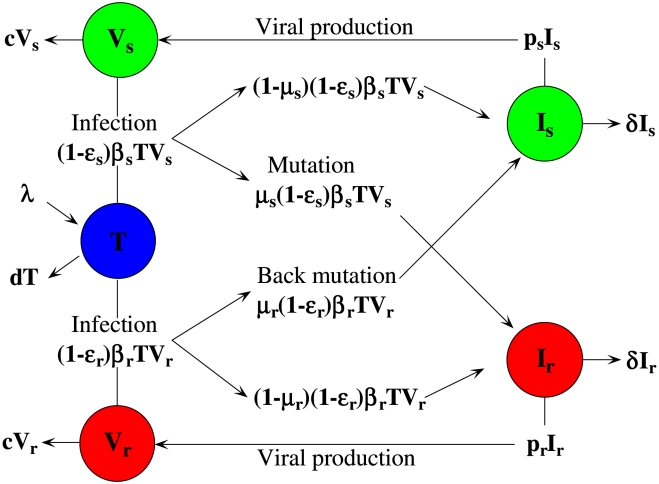
Schematic diagram of the viral dynamic model.

A schematic diagram of the model is shown in Fig. 1. The model contains five variables: uninfected target cells, *T*, cells infected by ENF-sensitive virus, *I_s_*, cells infected by ENF-resistant virus, *I_r_*, ENF-sensitive virus, *V_s_*, and ENF-resistant virus, *V_r_*. The model assumes that target cells are produced at a constant rate, *λ*, and die at rate *dT*. ENF-sensitive virus infects target cells to produce infected cells, *I_s_*, at rate *β_s_TV_s_*, among which a fraction *μ_s_β_s_TV_s_*, become ENF-resistant during the process of reverse transcription of viral RNA to DNA due to mutation at rate *μ_s_*. Similarly, the infection by ENF-resistant virus produces infected cells, *I_r_*, at rate *β_r_TV_r_*, with a fraction *μ*_r_ *β*_r_ *TV_r_* undergoing backward mutation to the drug sensitive strain at rate *μ*_r_. Cells infected by ENF-sensitive and ENF-resistant virus produce new virions at rates *p_s_I_s_* and *p_r_I_r_*, and die at rates *δI_s_* and *δI_r_*, respectively. Both viruses are cleared at the same rate *c* per virion. Whether the V38A mutation in gp41 affects viral production remains unclear. For simplicity, we assume *p_s_* = *p_r_*, and describe the resistance-associated fitness loss only by a reduced infectivity rate, i.e., *β_r_* = (1−*α*) *β_s_*, where the fitness cost of the mutant virus, *α*, satisfies 0≤*α*≤1. ENF is a fusion inhibitor and reduces infection of target cells by free virus. We assume *ε_s_* and *ε_r_* are the efficacies of ENF against ENF-sensitive and ENF-resistant virus, respectively, with 0≤*ε_s_*, *ε_r_*≤1.

In the patient data we analyze the populations of both drug-resistant and drug-sensitive virus always remain high (above 2.8 log_10_ HIV RNA copies/ml). Thus, stochastic effects would not be significant and we formulate the model as a deterministic model – a standard two-strain viral dynamic model – similar to the ones in [20], [21]. The model is described by the following differential equations:
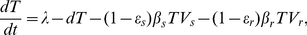
(1)

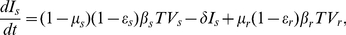
(2)

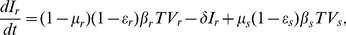
(3)

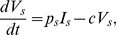
(4)

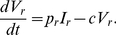
(5)


As measured by Ki-67 antigen expression, only a small percentage of CD4^+^ T cells in peripheral blood appear to be activated into proliferation and hence are preferred targets for HIV-1 infection [22]. Therefore, we take only a fraction of the total CD4 count, i.e. the activated cells, as targets for HIV-1 infection and estimate this fraction. The total CD4^+^ T cell count is assumed to be given by (*T+I_s_+I_r_*)/*a*, where *a* denotes the fraction of CD4^+^ T cells that are activated. In principle, *a* could be time-varying or in particular depend on the CD4^+^ T cell count [22]. However, the CD4 count of the patients in this study always remains below 200/µl, and according to the relationship between CD4 count and activated cell percentage given in [22], a 10-fold change in CD4 count (from 20 to 200/µl) causes only a minor change in activation percentage (from 8.6% to 10.4%). Therefore, for our study we felt it reasonable to assume *a* is constant.

We note that in the study we analyze [13], ENF is given in combination with other drugs, the infection rates *β_s_*, *β_r_* and the virus production rates *p_s_*, *p_r_* that we estimate include the effects of the other drugs in the background regimen. However, since the background regimen was failing to suppress HIV replication, these effects may be minimal. Moreover, the data have taken only V38A mutants into account with other mutants being included in the “wild-type”. Therefore ENF efficacy against wild-type, *ε_s_*, in our model also incorporates the possible reduction in efficacy due to other mutants included in the wild-type. Further, loss of V38A mutation at rate *μ_r_*, can lead to any of a variety of viral variants that we include in the drug sensitive population. Lastly, virus variants carrying the V38A mutation may also carry other mutations, such as compensatory mutations or other drug resistance mutations, which may affect the fitness of the drug-resistant population as well as its level of drug resistance.

We note that there is loss of some free virus due to the infection of target cells as virus must enter a cell in order to infect. To incorporate this effect, one can add the terms −*(1−ε_s_)β_s_TV_s_* and −*(1−ε_r_)β_r_TV_r_* to Eqs. (4) and (5), respectively. For the measured range of *T* in the subjects considered here, and the estimates of *β_s_* and *β_r_* determined below, *β_s_T* and *β_r_T* are <0.05 d^−1^ which is ∼500 times lower than the viral clearance rate *c* (23 d^−1^), indicating that virion loss due to infection will have negligible effect on the viral dynamics compared to the term −*cV*. We confirmed this by fitting the model with the terms −*(1−ε_s_)β_s_TV_s_* and −*(1−ε_r_)β_r_TV_r_* in Eqs. (4) and (5), respectively, in which we found almost no change in parameter estimates. Therefore, we neglected virion loss due to infection and left only the viral clearance term (−*cV*) in the *V* equations.

### Parameter Estimation

The dynamics of free virus is typically fast in comparison with that of infected cells [23]–[25]. Therefore, we assume a quasi-steady state, which from Eqs. (4) and (5) provides *V_s_* = *(p_s_/c)I_s_* and *V_r_ = (p_r_/c)I_r_*. This simplifies the model leaving only equations for *T*, *I_s_* and *I_r_*. Further, we set *I_s_*(0) = (*c*/*p_s_*)*V_s_*(0) and *I_r_*(0) = (*c*/*p_r_*)*V_r_*(0) for data fitting as well as all simulations, where *V_s_(0)* and *V_r_(0)* are determined by direct measurement at the start of interruption or the start of ENF re-administration.

As measured by Mohri et al. [26], we take the uninfected CD4^+^ T cell death rate *d* = 0.01 day^−1^. Recent estimates show that the virion clearance rate constant, *c*, varies between 9.1 day^−1^ and 36 day^−1^, with an average of 23 day^−1^ [25], [27]. Therefore, we take *c* = 23 day^−1^.

During ENF interruption, we estimate the parameters *λ* (target cell recruitment rate), *β_s_* (drug sensitive virus infection rate), *μ_s_* (forward mutation rate), *μ_r_* (backward mutation rate), *α* (fitness cost of ENF-resistance), *p_s_* (production rate of drug sensitive virus), *δ* (infected cell death rate), *T_0_* (initial uninfected target cell concentration) and *a* (fraction of CD4^+^ T cells that are activated) by fitting the model to the ENF-sensitive viral load, the ENF-resistant viral load and the CD4 count data simultaneously for each patient. Since fewer data points are available during re-administration of ENF, we fix some parameters at the values obtained by estimation during ENF interruption; and only estimate *ε_s_* (ENF efficacy against the sensitive strain), *ε_r_* (ENF efficacy against the resistant strain), *λ*, *T_0_* and *a*. We also fitted the data during ENF interruption and ENF re-administration allowing the initial concentrations of drug sensitive and drug resistant viruses to be free parameters, but the fit could not be improved.

We solved Eqs. (1)–(5) numerically using the Runge-Kutta 4 algorithm in Berkeley Madonna [28]. We also used it to obtain the best-fit parameters via a nonlinear least squares regression method. The predicted log_10_ values of the ENF-sensitive and ENF-resistant viral loads and the CD4 count for each patient were fit to the corresponding log-transformed data. Of note, to avoid the difficulty of assigning different weights to the viral loads and CD4 counts in the objective function being minimized, and give equal importance to all the widely varied values in the data set, we fitted the data in the log-scale rather than linear-scale for both viral loads and CD4 count. Finally, for each best fit parameter estimate, we provide a 95% confidence interval (CI) using 200 bootstrap replicates [29], which we performed in MATLAB.

## Results

### Viral Dynamic Parameters during ENF Interruption

**Figure 2:**
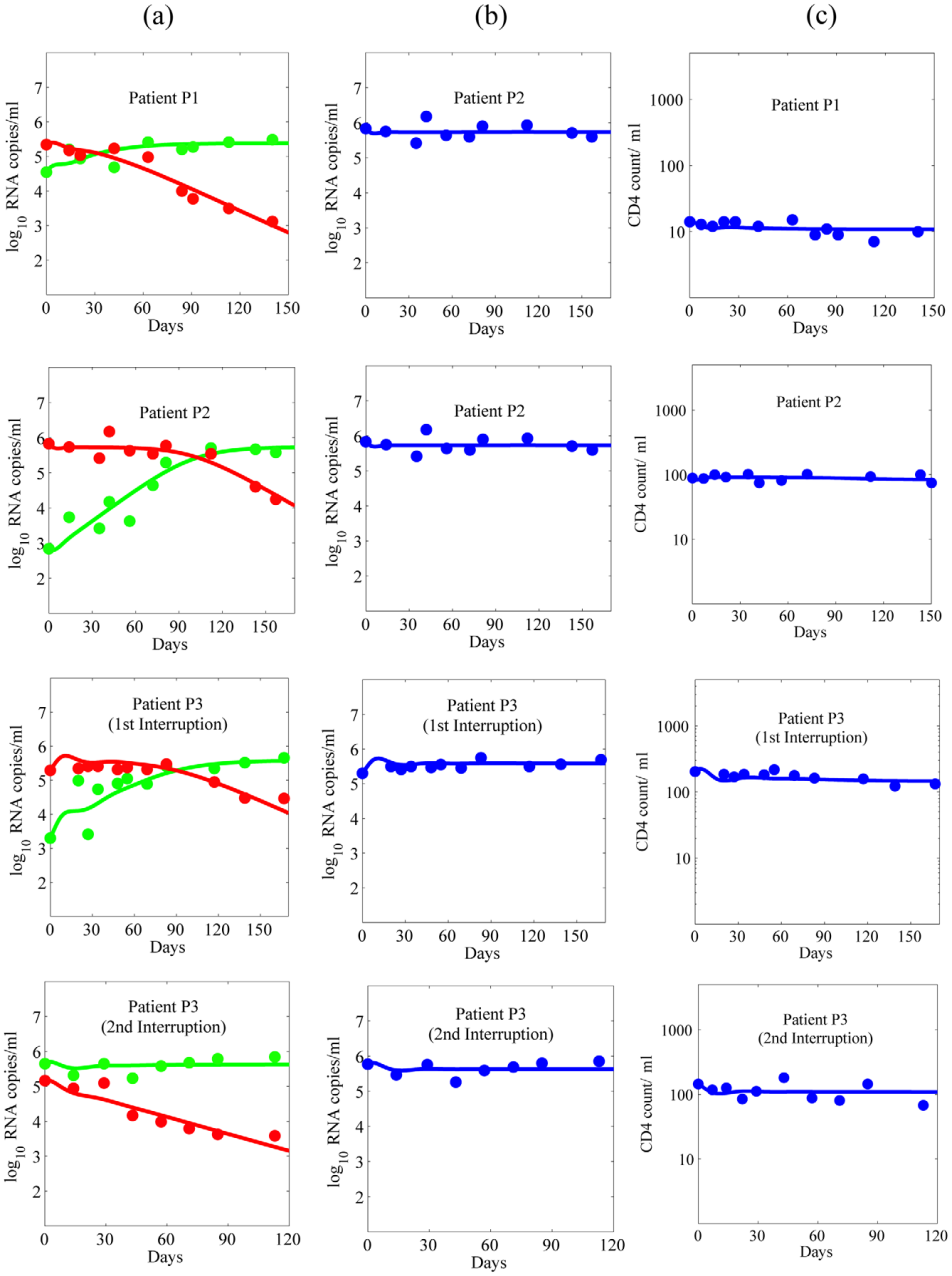
Dynamics during ENF interruption. (a) Wild-type (green) and ENF-resistant (red) HIV-1, (b) the total plasma viral load (blue), and (c) CD4^+^ T cells (blue), predicted by the model using estimated parameters (solid curve) and experimentally observed data (•).

The estimated viral dynamic parameters during ENF interruption along with their mean and sample standard deviation, and their 95% confidence interval are summarized in Table 1 and Table 2, respectively. Using the estimated parameters, we found the predictions of the model agree well with the data for each of the study participants (Fig. 2). All the parameters are approximately the same for two ENF-interruptions in P3, suggesting that the viral dynamic parameters remain stable over time.

**Table 1:** Estimated parameters during ENF interruption.

Patient	*λ*	*β_s_*	*μ_s_*	*μ_r_*	*α*	*p_s_*	*δ*	*T_0_*	*α*
	(cells/ml/day)	(10^−7^) (/ml/day)	(10^−5^)	(10^−5^)		(virions/infected cell)	(1/day)	(log_10_)	
P1	343	10.26	2.49	1.80	0.18	4770	0.28	4.16	0.23
P2	1059	9.09	1.78	1.30	0.20	3296	0.28	4.94	0.07
P3 (1^st^)*	911	4.05	2.30	1.80	0.16	2742	0.27	5.33	0.06
P3 (2^nd^)*	845	4.87	2.40	2.00	0.13	3704	0.31	5.16	0.06
**Mean**	790	7.07	2.24	1.73	0.17	3628	0.29	4.90	0.11
**SD**	311	3.07	0.32	0.30	0.03	857	0.02	0.52	0.08

*: For patient P3, there are two interruptions of ENF.

**Table 2:** The 95% confidence intervals obtained by 200 bootstrap replicates for parameter estimates in Table 1.

Patient		*λ*	*β_s_*	*μ_s_*	*μ_r_*	*α*	*p_s_*	*δ*	*T_0_*	*α*
		(cells/ml/day)	(10^−7^) (/ml/day)	(10^−5^)	(10^−5^)		(virions/infected cell)	(1/day)	(log_10_)	
P1	Lower	322	9.1	2.32	0.92	0.17	4582	0.27	4.14	0.22
	Upper	394	12.8	5.49	5.08	0.20	4856	0.33	4.18	0.25
P2	Lower	997	8.12	0.82	0.77	0.15	3014	0.27	4.93	0.06
	Upper	1335	14.17	5.32	3.83	0.21	3944	0.40	4.94	0.08
P3	Lower	879	4.01	1.12	1.00	0.16	2217	0.19	5.30	0.06
	Upper	982	6.00	5.33	4.81	0.24	2942	0.33	5.33	0.07
P4	Lower	810	3.89	2.10	1.21	0.12	3353	0.29	5.12	0.05
	Upper	1108	7.45	5.42	5.48	0.15	3787	0.36	5.16	0.08

We estimated the rates of forward and backward mutation as 2.24±0.32×10^−5^ and 1.73±0.30×10^−5^, respectively. Even though the backward mutation rate is slightly lower than the forward mutation rate, early after ENF interruption the ENF-resistant virus population is significantly larger than the ENF-sensitive virus population, and consequently the amount of backward mutation during the early post ENF interruption is usually higher than the amount of forward mutation. Although there is a continued evolution in gp41 after ENF interruption, our results show that the rate of on-going evolution including backward mutation accumulation is not sufficient to explain the rapid waning of ENF-resistant virus. For example, during the first week (month) post interruption, the contribution of ongoing evolution and backward mutation to the loss of cells carrying a drug-resistant proviral genome is only about 0.2 (0.4) cells per ml, which corresponds to the loss of 26 (70) drug resistant virions per ml per week (month). Since the contribution of these de-novo mutations is small, we also fitted the data using the model without de-novo mutation, i.e., *μ_s_* = *μ_r_* = 0, and found that the changes in estimated parameter values lie within a range of 0–6%. As the loss of resistant virus due to backward mutation is negligible, we find the fitness cost, i.e., the reduction of the infectivity of the resistant virus compared to the wild-type virus, plays a more important role in the decay of ENF-resistant virus and the increase of ENF-sensitive virus. Fitting our model to the data suggests that ENF-resistant virus is 17±3% less fit (i.e., *α* = 0.17±0.03, Table 1) than ENF-sensitive virus in the absence of ENF. This fitness loss is consistent with the results in Marconi et al. [13], although they obtained a higher estimate of the relative fitness cost (25–65%) using a different fitness estimation method.

Our estimates of the virion production rate, *p_s_* = *p_r_* = 3628±857 virions day^−1^, the infected cell death rate, *δ* = 0.29±0.02 day^−1^, and the sensitive virus infection rate, *β_s_* = 7.1±3.1×10^−7^ ml^−1^ day^−1^ (Table 1), are approximately consistent with the estimates 1427±2000 virions day^−1^, 0.37±0.19 day^−1^ and 11.8±14×10^−7^ ml^−1^ day^−1^, respectively, in Stafford et al. [30]. However, the estimate of *δ* is much smaller than that in some other studies [31]. We also estimated the uninfected cell recruitment rate *λ* = 790±311 cells ml^−1^ day^−1^. Our estimate of *a* suggests that 11% of CD4^+^ T cells are activated, consistent with the finding that ∼10% of CD4^+^ T cells in peripheral blood are Ki-67^+^ in the patients with CD4 count less than 200 cells/µl [22].

### Effectiveness of ENF Re-Administration after ENF-Interruption

**Figure 3:**
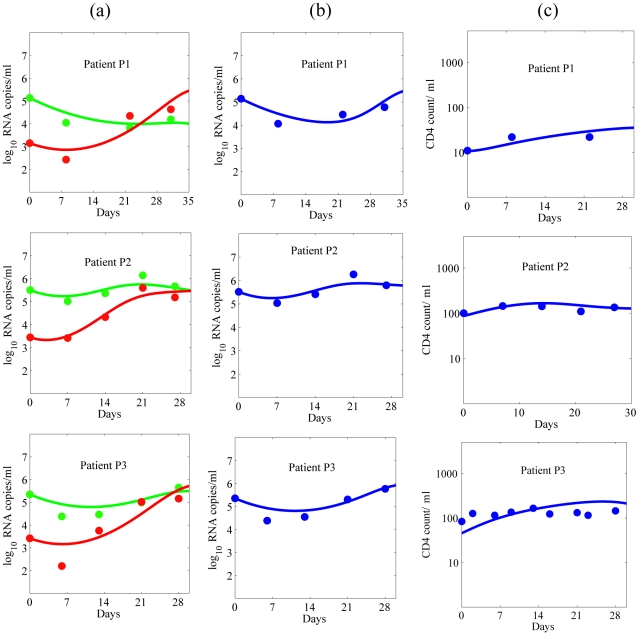
Dynamics during ENF re-administration after interruption. (a) wild-type (green) and ENF-resistant (red) HIV-1, (b) the total plasma viral load (blue), and (c) CD4^+^ T cells (blue), predicted by the model using estimated parameters (solid curve) and experimentally observed data (•).

After ENF interruption, ENF was re-administered to the study subjects for 4 weeks while keeping the same “background” regimen. During this re-administration of ENF, we estimated the ENF efficacies against sensitive and resistant viruses, *ε_s_* and *ε_r_*. Estimated values and their 95% confidence intervals are summarized in Table 3 and Table 4, respectively. Comparisons of model predictions with the patient data are shown in Fig. 3.

**Table 3:** Estimated parameters during ENF re-administration after interruption.

Patient	*ε_s_*	*ε_r_*	*λ*	*T_0_*	*α*
			(cells/ml/day)	(log_10_ cells/ml)	
P1	0.71	0.27	206	4.03	0.15
P2	0.67	0.36	1106	4.72	0.07
P3	0.58	0.25	949	4.66	0.08
**Mean**	0.66	0.29	754	4.47	0.10
**SD**	0.06	0.06	481	0.38	0.04

**Table 4:** The 95% confidence intervals obtained by 200 bootstrap replicates for parameter estimates in Table 3.

Patient		*ε_s_*	*ε_r_*	*λ*	*T_0_*	*α*
				(cells/ml/day)	(log_10_ cells/ml)	
P1	Lower	0.66	0.14	192	3.96	0.11
	Upper	0.83	0.28	207	4.05	0.16
P2	Lower	0.63	0.30	1005	4.69	0.07
	Upper	0.72	0.47	1303	4.73	0.08
P3	Lower	0.50	0.10	869	4.62	0.07
	Upper	0.75	0.29	1023	4.66	0.09

Our estimates indicate that ENF re-administered following interruption is 66±6% effective in reducing infection by ENF-sensitive virus, while the effectiveness is reduced to 29±6% in reducing infection by ENF-resistant virus. This indicates that ENF-resistant variants still remain partially sensitive to ENF even though they have reduced susceptibility. We note that the efficacy of ENF against drug sensitive virus obtained here is a minimal estimate as it might have included the reduction of efficacy due to inclusion of other mutant virus in the drug sensitive virus data. Other estimated parameters during ENF re-administration (Table 3) are more or less the same as those estimated during ENF interruption (Table 1). The continued activity of ENF against the drug-resistant virus is supported by the apparent immediate albeit transient and small increase in plasma HIV RNA levels observed when ENF was interrupted in a larger cohort of individuals (see Figure 1 in [11]).

### Plasma Viral Load

Despite the difference in replication capacity and changes in the proportion of ENF-sensitive and ENF-resistant viruses (Fig. 2a), the total plasma viral load remains approximately the same during ENF interruption (Fig. 2b). The plasma viral load also remains unchanged during ENF re-administration (Fig. 3) except for a nominal transient post-readministration suppression followed by a rebound. This raises a question: what determines the plasma viral load?

**Figure 4:**
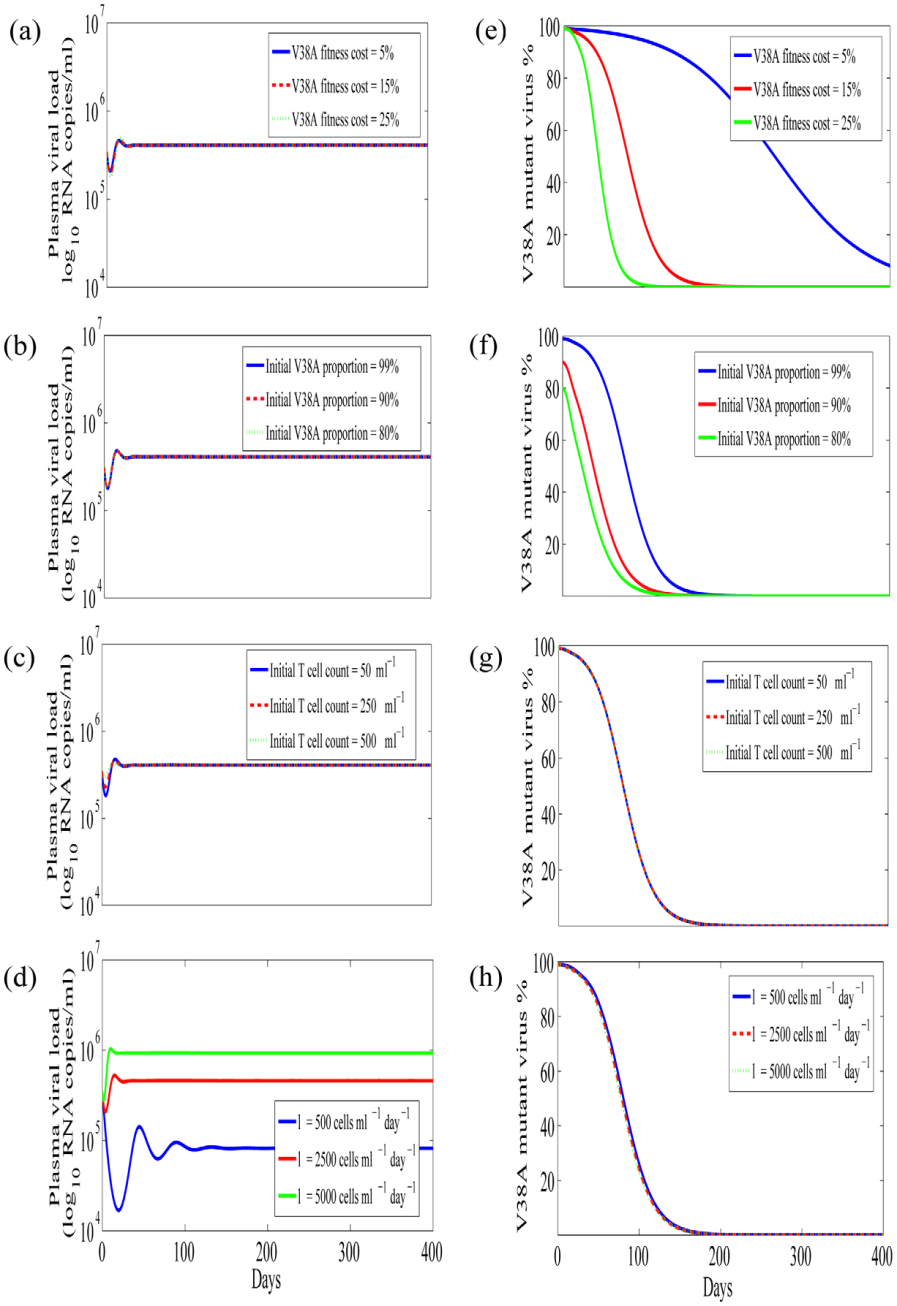
Simulation results during ENF interruption. Plasma HIV-1 RNA level (a–d), and V38A mutant virus proportion (e–h), for different V38A mutant virus fitness costs (a,e), initial V38A mutant virus proportion (b,f), initial T cell counts (c,g) and T cell source rate (d,h). Parameters used are the average values of P1, P2, P3 in Table 1.

**Figure 5:**
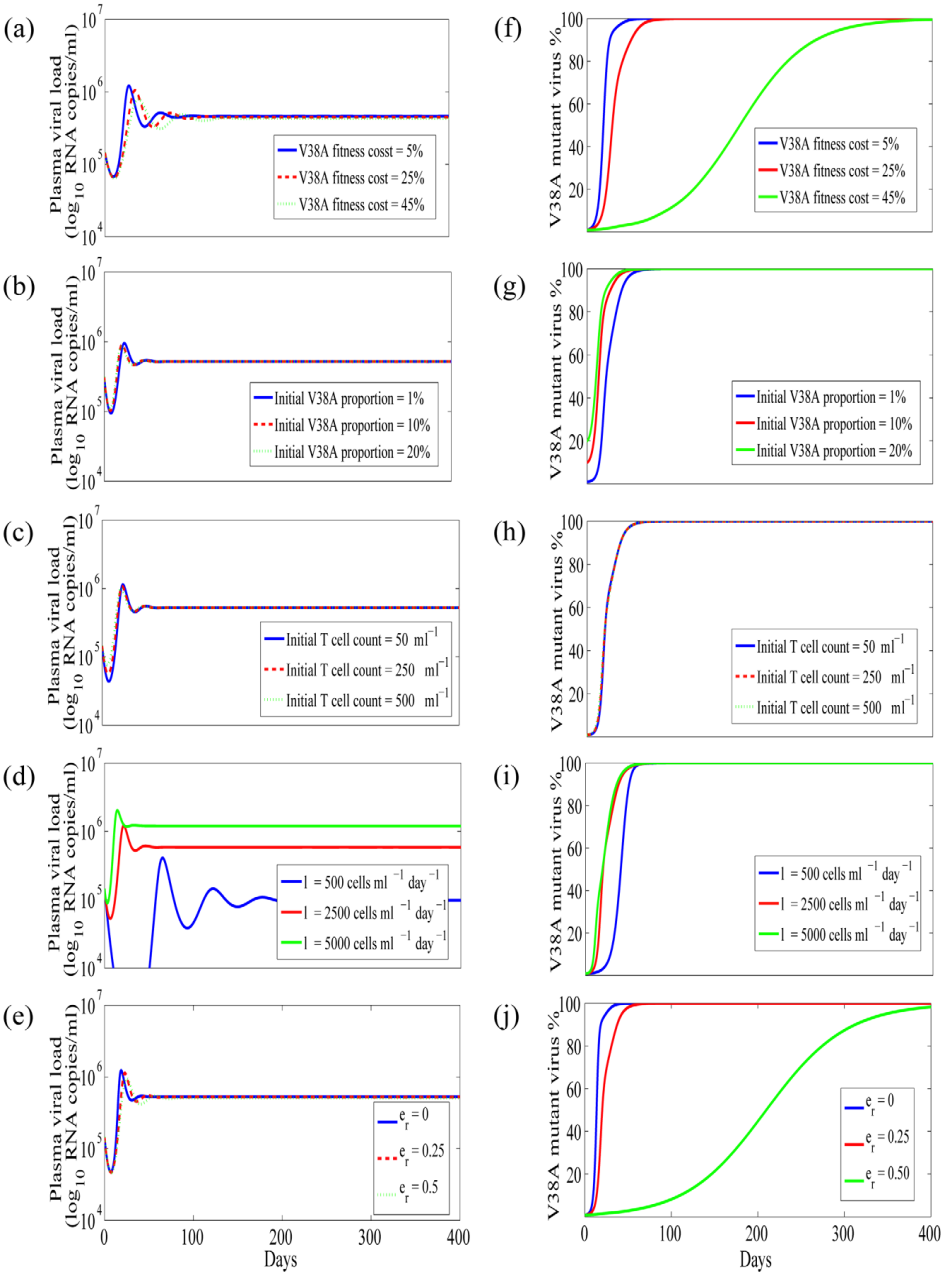
Simulation results during ENF re-administration. Plasma HIV-1 RNA level (a–e), and V38A mutant virus proportion (f–j), for different V38A mutant virus fitness costs (a,f), initial V38A mutant virus proportion (b,g), initial T cell counts (c,h), T cell source rate (d,i) and ENF efficacy against ENF-resistant virus (e,j). Parameters used are the average values in Table 3.

We first studied the effect of ENF-resistant virus fitness cost on plasma viral load. In Figs. 4a and 5a, we show the plasma viral load obtained from our model for different fitness costs during ENF interruption and ENF re-administration, respectively, with other parameter values held to their estimated values. When we varied the fitness cost from 5 to 50% we did not find any observable change in plasma viral load. This suggests that the fitness cost has a minor role in determining the total viral load. We next studied the effect of different initial proportions of the mutant virus at the time of ENF interruption (Fig. 4b) and ENF re-administration (Fig. 5b). The initial proportion of ENF-resistant virus does not seem to have any effect on plasma viral load either.

From the model we can calculate the steady state level of infected cells, 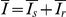, which given our assumption that *p_s_* = *p_r_* = *p*, is proportional to the total viral load, 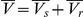, i.e. 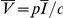, where an over-bar denotes a steady state value. As the resistant virus population decays to a low level during ENF interruption, the net effect of backward mutation on the steady state is negligible. Therefore, we neglect backward mutation and obtain the following expression for the steady state total viral load, 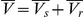:
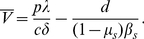
(6)Note that 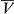 is independent of the fitness cost, *α*.

Similarly, during ENF re-administration we neglect the forward mutation rate as the sensitive virus replication is largely inhibited, and obtain the steady state total viral load in the presence of ENF, 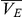, as
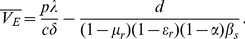
(7)In this case, the total viral load depends upon the fitness cost, *α*. However, using our estimated parameters (Table 3), the second term on the right hand side is ∼20-fold smaller than the first term and hence the effect is negligible as seen in Fig. 5a. Therefore, the fitness cost again does not have any effect on setting the total viral load.

We next studied the effect of target cells on the total viral load. We considered two approaches: one by changing the initial target cell level and another by changing the recruitment rate of target cells. We did not observe any effect of the initial target cell level on the total viral load during ENF interruption (Fig. 4c) or re-administration (Fig. 5c).

Marked differences in the level of plasma viral load is seen when the target cell recruitment rate, *λ*, changes while keeping all other parameters fixed, during both ENF interruption (Fig. 4d) and re-administration (Fig. 5d). After early transient changes in viral load upon ENF interruption, the plasma viral load level remains relatively constant during the interruption with the level related to the target cell source rate *λ*. A similar result is found during ENF re-administration except that it takes longer to initially stabilize the viral load level during ENF re-administration than during ENF interruption. While we demonstrated the dependence of the viral load on *λ* (Figs. 4d and 5d), the level of plasma viremia can also be seen by simulation to depend on *p*, *c* and *δ*. This is also supported by the analytical expressions (6) and (7) for the steady state level of total virus, which to a good approximation are equal to, *pλ/(cδ)*, during ENF interruption and re-administration.

### The Target Cell Level

**Figure 6:**
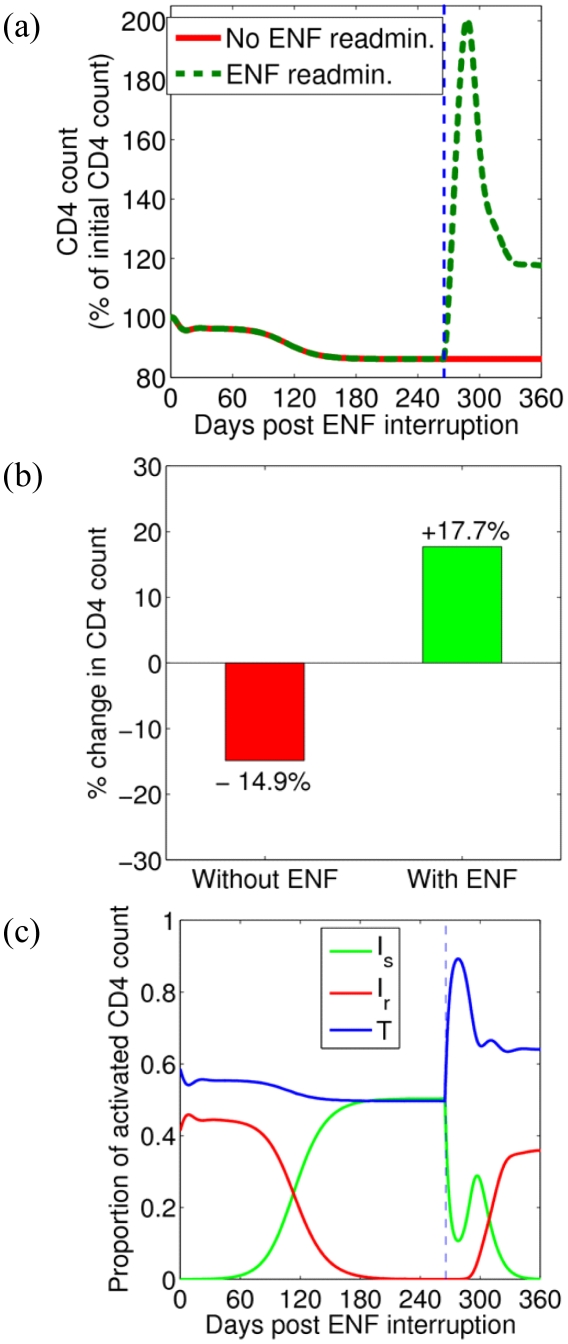
CD4 count and the proportion of uninfected and infected cells. (a) Predicted temporal variation of the CD4 count during ENF interruption (red) and with ENF re-administration (green). Parameters used are the average values in Tables 1 and 3. The vertical dashed line indicates the time of ENF re-administration. (b) Bar diagram showing the percentage change of CD4 count at the end of one year without ENF (red) and at the end of 3 months with ENF re-administration (green). (c) Change over time of the proportion of uninfected cells, *T* (blue), cells infected with drug-sensitive virus, *I_s_* (green), and cells infected with drug-resistant virus, *I_r_* (red). The vertical dashed line indicates the time of ENF re-administration.

The changes over time of the CD4 count, and of the proportion of uninfected cells, cell infected with sensitive virus, and cell infected with resistant virus are shown in Figs. 6a and 6c, respectively. After ENF re-administration, the proportion of uninfected cells increases, reaches a peak and then decays to a steady-state level higher than the level before ENF re-administration. In a study by Deeks et al. [11] on a larger cohort of individuals, the subjects received an ENF-based regimen (the same as the one received by individuals in this study) for 34 weeks (approximately the same period as in our study) followed by the interruption of ENF. During a screening period of 4 weeks just before the interruption began, they found a negligible change in CD4^+^ T cell counts (mean change: 0.13 cells/µl/week) suggesting that steady state was reached by the end of this long-term treatment. They also observed the steady state T cell level after a long period of ENF interruption. Below we calculate from our model the steady state level of uninfected CD4^+^ T cells to understand how the uninfected target cell level differs between long-term ENF interruption and long-term ENF re-administration.

The steady state level of target cells during ENF interruption, *T_E_*, and ENF re-administration, 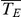, can be calculated from our model and are given by
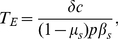
(8)

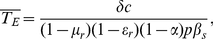
(9)respectively. Before ENF is re-administered, *ε_r_* = 0, as no drug is present. After drug is given, *ε_r_*>0 and Eq. (8) shows that the target cell level should increase. Furthermore, in addition to the efficacy of the drug against resistant virus, *ε_r_*, the fitness cost, *α*, also contributes to the maintenance of a higher level of uninfected target cells during ENF re-administration. In fact, even if the drug is completely ineffective against resistant virus (i.e., *ε_r_* = 0), and the viral load is approximately equal during both ENF interruption and ENF re-administration as shown above, HIV infected patients with ENF re-administration will still have a higher uninfected target cell level due to the fitness loss of resistant virus (i.e., for *α*>0). Above we showed that during re-administration the total viral load, and hence the total number of infected cells also stays approximately constant. Hence the CD4^+^ T cell count, which includes both uninfected and infected CD4^+^ T cells, is expected to increase with the increase in target cells. This is an important result as it shows that even though the resistant virus becomes dominant during ENF re-administration (Fig. 3), the CD4^+^ T cell count should increase, which represents an immunologic benefit to patients.

### Competition between Two Strains

Before ENF interruption, the ENF-resistant viral load is on average 100-fold higher than ENF-sensitive viral load. After ENF interruption the proportion of ENF-resistant virus decreases (Fig. 2) and after several weeks ENF-sensitive virus becomes dominant. According to our simulations, the time it takes for ENF-sensitive virus to take over the viral population mainly depends upon the fitness cost (Fig. 4e) and the initial proportion of ENF-resistant virus (Fig. 4f). To look at this more closely, we simplify the problem by neglecting mutation and by assuming the target cell level remains constant, i.e. we assume 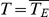, the steady state target cell in the presence of drug (i.e. before the interruption). This results in a system of two linear differential equations in *I_s_* and *I_r_*. Solving these equations, we obtain the following expression for *r*(t) = *V_r_*(t)/*V_s_*(t), the ratio of the two strains:
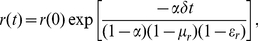
(9)where *r*(0)≈100, i.e. resistant virus is approximately 100-fold more plentiful than sensitive virus. As time off therapy increases the level of resistant virus falls and *r*(t) decreases. When *r*(t)<1, the sensitive virus is the dominant strain. The time, *t_θ_*, for the proportion of resistant virus to reach *r*(*t_θ_*) during ENF interruption is
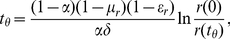
(10)As indicated by the above expression, and as seen in Figs. 4e and 4f, an increase in the fitness cost, *α*, causes the ENF-sensitive virus to be dominant sooner, while an increase in initial ENF-resistant virus proportion, *r*(0), results in a longer time for the ENF-sensitive virus to be dominant. Varying the T-cell count at the time of interruption from 50 to 250 or 500 µl^-1^ or increasing the T cell source rate, *λ*, does not significantly impact the proportion of ENF-resistant virus (Figs. 4g and 4h).

We also studied the competition of the virus populations during ENF re-administration (Figs. 5f–j). Following ENF re-administration ENF-resistant virus reemerges rapidly and attains the proportion 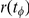 in an approximate time 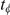 (obtained as in ENF interruption case above) given by
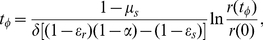
(11)For the parameters in Tables 1 and 2, the virus population changes more rapidly during ENF re-administration than during ENF interruption, and the time for the virus population to become dominated by resistant virus, i.e. for *r*>1, mainly depends upon the combined effect of fitness cost and efficacy of ENF against the ENF-resistant virus. If ENF is sufficiently effective against ENF-resistant virus or if the fitness cost is sufficiently high, the turnover is significantly delayed. In addition to ENF efficacy and the fitness cost, there appear to be nominal effects of the initial ENF-resistant virus proportion, and target cell generation rate on the turnover of the virus population during ENF re-administration (Figs. 5g,i).

### Benefits of ENF Re-Administration Following an Interruption

Despite the resistance to ENF, re-administration of ENF might provide some benefits if ENF has partial activity against resistant virus. Using our model to study this, we found re-administration of ENF results in transient nominal viral suppression for about 2 weeks followed by a rapid rebound in plasma HIV-1 RNA level and then attainment of a steady state viral load higher than the initial viral load in about 7 weeks (Fig. 5e). This shows that ENF re-administration is not effective in suppressing plasma viral load. However, our model simulations show that re-administration of ENF helps in maintaining a higher CD4^+^ T cell level (Fig. 6a). After ENF re-administration, the CD4 count increases, reaches a peak and decays to a steady state level higher than the steady state level before the re-administration. While the CD4^+^ T cell count decreases by 15% in the absence of ENF, re-administration of ENF results in an increase of the CD4^+^ T cell count by 18% over the treatment period of 3 months (Fig. 6b), which can be clinically significant. This gain of about 35% in the CD4 count due to ENF re-administration predicted by our model is consistent with a ∼36.8% increase in CD4 count (from 95 cells/µl to 130 cells/µl) during ENF-treatment observed in a study of 25 individuals [11]. According to our model, this increase is observed because during re-administration of ENF, ENF-sensitive virus is replaced by ENF-resistant virus that has less ability to infect CD4^+^ target cells (Fig 6c). Therefore, there appears to be an immunological benefit, i.e., achieving a higher CD4^+^ T cell count, in patients taking ENF, even though they might suffer virologic failure due to the emergence of resistance. The level of CD4^+^ T cells increases as fitness cost or/and the efficacy of ENF against ENF-resistant virus increases because an increase in fitness cost or/and efficacy further decreases the infectivity of resistant virus.

## Discussion

The impact of antiretroviral drug-resistance on viral load, CD4^+^ T cell counts and clinical outcomes is complex. Although the emergence of resistance to protease inhibitors and reverse transcriptase inhibitors clearly affects viral fitness (as defined in vitro and in vivo) [2]–[5], [32], its impact on viral load and CD4^+^ T cell counts is unclear. At comparable plasma viral loads, drug resistant HIV can be associated with more sustained CD4^+^ T cell gains and reduction of the risk of morbidity and mortality [2], [4], [5], [32] than wild-type (drug-sensitive) HIV. To understand the mechanism for this apparent beneficial effect on immunologic and clinical outcomes independent of viremia, we use ENF resistance as a “probe” to explore the impact of fitness on viral and immunologic dynamics in vivo. Although the data linking ENF resistance to viral load, CD4 and clinical outcomes is limited, the preliminary data that does exist is consistent with the more extensive literature pertaining to protease inhibitor resistance. Specifically, despite the emergence of ENF-resistant mutations, CD4^+^ T cell counts have been observed to increase during therapy as the ENF resistant virus with less capacity to infect T cell replaces the ENF-sensitive virus. A large prospective study has recently been completed in which ENF was given as a “pulse” to determine if the expansion of ENF resistance positively affects CD4^+^ T cell counts. Preliminary data from 3 individuals has previously been published [13]. Given the richness of this data-set, we developed a mathematical model to study the benefits of ENF re-administration after interruption of therapy due to virological failure.

Interruption of ENF after the emergence of ENF resistance results in a rapid decay of the resistant variant [13]. One of the key questions is what factors play a role in the waning of the ENF-resistant virus and in determining the time for the ENF-sensitive virus to become dominant. Similar questions arise for the period of ENF re-administration in which ENF-resistant virus rapidly increases and takes over the ENF-sensitive virus. Moreover, despite the rapid turnover of the virus population, the plasma HIV-1 RNA level remains unchanged during ENF interruption raising a question of what determines the plasma viral load. In this study, we took advantage of mathematical models to address these issues.

Our model, which describes the dynamics of ENF-sensitive virus, ENF-resistant virus, target cells, cells infected by ENF-sensitive virus and cells infected by ENF-resistant virus, includes the fitness cost of ENF-resistant virus as well as forward and backward mutations. The model was used to fit data concerning the level of ENF-sensitive viruses, V38A ENF-resistant viruses and the CD4^+^ T cell count from HIV-1 infected patients, who underwent ENF interruption and subsequent re-administration while continuing to receive the other drugs in their regimen [13]. The data fitting during ENF interruption allowed us to estimate the forward mutation rate, the backward mutation rate and the fitness cost of the ENF-resistant virus along with the virus production rate, the infected cell death rate, the infection rate, the source rate of the target cells and the fraction of T cells that were target cells. Moreover, the data fitting allowed us to estimate the ENF efficacy against ENF-sensitive and ENF-resistant viruses during ENF re-administration.

Our parameter estimates, model analysis and numerical simulations produced several interesting observations. First, the magnitude of the backward mutation rate of V38A is approximately the same as that of the forward mutation rate. This indicates that ongoing viral evolution might have some contributions to the loss of ENF-resistance during ENF interruption, supporting the results of phylogenetic analysis in Kitchen et al. [9]. However, we observe that backward mutation barely contributes to the loss of drug resistant viruses (26 virions/ml in the first week and 70 virions/ml in the first month post interruption), and thus is not sufficient to achieve the rapid waning of ENF-resistant viruses observed when therapy is interrupted. Outgrowth of the wild-type virus with a fitness advantage in the absence of drug is a more plausible explanation.

Second, we found that the fitness cost of ENF-resistant mutations has a major role in the loss of ENF-resistant virus and the turnover of the virus population during ENF interruption. We estimated that the ENF-resistant virus is 17±3% less fit than the ENF-sensitive virus. This reduced fitness of V38A ENF-resistant virus agrees with the experimental finding of reduced fitness of the V38A mutant virus compared to wild-type virus in vitro [1]. Our simulations and analysis showed that the time needed for the sensitive virus to dominate the resistant virus during ENF interruption is mainly determined by the combined effect of fitness cost and the initial ENF-resistant virus proportion. Not surprisingly, the higher the fitness cost, the shorter the turnover time; and the higher the initial ENF-resistant virus proportion, the longer the turnover time (Fig. 4e,f). During ENF re-administration, the ENF efficacy against ENF-resistant virus also plays an important role in determining the time for the resistant virus to outcompete sensitive virus. A higher ENF efficacy against ENF-resistant virus results in a longer turnover time (Fig. 5j). Interestingly, there is a negligible effect of the target cell level on determining the turnover time of the virus population (Figs. 4g,h and 5h,i).

Third, we found that the fitness cost and the initial proportion of the ENF-resistant virus do not have any observable role in defining plasma HIV RNA levels. Neither does ENF efficacy during ENF re-administration contribute to setting plasma viral levels. The plasma viral RNA level is determined mainly by the target cell generation rate, *λ*, the virus production rate, *p*, the infected cell death rate, *δ*, and the virus clearance rate, *c*. A higher value of *p* or *λ* and/or a lower value of *δ* or *c* give rise to a higher plasma HIV RNA level.

Our next observation is related to the advantage or disadvantage of re-administrating the drug following an interruption. Once the drug is re-administered, the resistant virus rapidly reemerges and becomes dominant over the sensitive virus. This indicates a strong selective pressure of the drug on the virus population. The turnover rate of the virus population is more rapid during the drug re-administration than during the drug interruption. This shows that the advantage of the drug-resistant virus over drug-sensitive virus during the drug re-administration is greater than the disadvantage of the drug-resistant virus over the drug-sensitive virus during the drug interruption. The rapid reemergence of the resistant virus also indicates the persistence of actively replicating resistant virus as suggested in [33], [34]. Our parameter estimates indicate that ENF when re-administered is ∼29% effective against ENF-resistant virus and ∼66% effective against ENF-sensitive virus. This supports the antiviral activity against ENF-resistant viruses observed previously [11]. After re-administration of ENF, our model predicts a small transient suppression of viral load followed by a rebound to a higher plasma RNA level, consistent with the pattern shown by the data. This shows that the re-administration of ENF cannot suppress plasma virus for the long term.

One of the most interesting results demonstrated by our model is that despite sustained high levels of viral load, re-administration of ENF helps in maintaining a significantly higher level of CD4^+^ T cells (Figs. 5e and 6). During ENF re-administration patients can achieve more than 35% higher CD4^+^ T cell count over the period of 3 months compared to the same patients during ENF interruption (Fig. 6b). The CD4 count increase predicted by our model (∼35%) is consistent with the CD4 count gain in a study on a larger cohort of individuals [11] in which the subjects (with the same background regimen as in our study) maintained a 36.8% higher CD4 during ENF treatment than during ENF interruption. This immunologic benefit of ENF occurs even in the presence of high-level ENF resistance, in agreement with the findings in some individuals harboring viruses with ENF-resistance mutations under long-term ENF therapy [35]. This outcome on administering ENF can be explained by the presence of resistant viruses with a reduced infection capacity (Figs. 2 and 6c). The treatment alters the fitness of the virus by selecting the less fit resistant virus that helps in maintaining a higher CD4 count even though it is ineffective in suppressing the viral load. A similar effect has been seen in patients treated with reverse transcriptase and protease inhibitors such as proD30N, rtK65R and rtM184V [2]–[5]. The benefit of the drug is mediated by changes in both the fitness of the virus and the efficacy of the drug against resistant virus. The CD4^+^ T cell level during the drug re-administration increases as the efficacy of the drug against resistant virus increases or/and the fitness cost of resistant virus increases.

There are several limitations of this study. The results are based on limited data from only three subjects. Moreover, there are fewer data points available during ENF re-administration, which might produce more uncertainty in the results derived from ENF re-administration. To gain more confidence in the results obtained here, extensive studies with more data are necessary. We have considered the V38A mutant virus as a representative of all ENF-resistant viruses. However, there are many other mutant viruses, which may possess different fitness costs and different mutation rates. It should be noted that in the experiment only the proportion of V38A was measured, and so there might be other mutant virus resistant to ENF that would have been included in the ENF-sensitive viral load. A detailed quasi-species model, as in Murray and Perelson [36], may provide a better explanation of the phenomena and help in estimating a more accurate value of the T-cell benefit. However, such complex models require more detailed data sets in which the population levels of other members of the quasi-species are measured. Currently, such data is unavailable.

In summary, we have used mathematical models to help explain the viral dynamic properties of drug sensitive and resistant viruses in the presence and the absence of the drug ENF. Our results show that even though forward and backward mutations occur during therapy interruption, the primary factor leading to the loss of resistant virus during therapy interruption is the fitness cost of the resistant virus. In the presence of drug, the efficacy of drug against resistant virus is also one of the main factors determining dominance of the drug resistant virus in the population. More importantly, even though the drug is ineffective in suppressing plasma viral load due to the presence of resistant virus, our results support the concept that continued therapy may have a residual immunologic benefit by preserving peripheral blood CD4^+^ T cell levels.
